# Exploring the incidence of culturally responsive communication in Australian healthcare: the first rapid review on this concept

**DOI:** 10.1186/s12913-019-4859-6

**Published:** 2020-01-07

**Authors:** Carla Minnican, Gjyn O’Toole

**Affiliations:** 1 The University of Newcastle, Callaghan, Australia; 2 Discipline of Occupational Therapy, School of Health Sciences, Faculty of Health and Medicine, The University of Newcastle, Callaghan, 2308 Australia

**Keywords:** Cultural competency, Responsive communication, Allied health, Rapid review, Healthcare, Prevalence

## Abstract

**Background:**

Increasing diversity in Australia requires healthcare practitioners to consider the cultural, linguistic, religious, sexual and racial/ethnic characteristics of service users as integral components of healthcare delivery. This highlights the need for culturally appropriate communication and care. Indeed the Australian Government in various policies mandates culturally responsive communication. Therefore this paper aims to provide a brief overview of Australian healthcare literature exploring the components; prevalence and effects of this style of communication in healthcare.

**Methods:**

A rapid review was conducted using the knowledge to action evidence summary approach. Articles included in the review were those reporting on the components, prevalence and outcomes of culturally responsive communication in Australian healthcare, published in English between 2008 and 2018. Articles were reviewed using reliable critical appraisal procedures.

**Results:**

Twenty- six articles were included in the final review (23 qualitative studies; 2 systematic reviews; 1 mixed methods study). The literature indicates knowledge of the positive effects of culturally responsive communication in healthcare. It also highlights the disparity between the perceptions of healthcare practitioners and services users over the existence and components of culturally responsive communication in healthcare. The review identified a limited use of this style of communication, but rather a focus on barriers to culturally appropriate care, lacking an awareness of the importance of culturally responsive communication in this care.

**Conclusion:**

While literature suggests the importance and positive effects of culturally responsive communication, evidence suggests inconsistent implementation of this style of communication within Australian healthcare settings. This has implications for the outcomes of healthcare for the diverse population in Australia.

## Background

Australia is culturally diverse, with various spoken languages, countries of birth, and religious affiliations [1]. Increasing diversity requires healthcare providers to consider the cultural, linguistic, religious, sexual and racial/ethnic characteristics of service users as integral components of providing quality healthcare [2]. Every individual has a slightly different culture and culturally determined perspective affecting his or her understanding, expectations and styles of communicating [3]. Thus, every clinical encounter is potentially cross-cultural [4]. Cultural responsiveness within healthcare services has been seen to improve health outcomes, reduce health disparities and contribute to shaping the health-related values, beliefs and behaviours of marginalised communities [5–7]. Communication and cultural responsiveness are intrinsically linked [8], with research indicating that ineffective communication can contribute to misunderstandings, inadequate or negligent care, and inappropriate interventions [3, 9]. Effective cross-cultural communication is especially important for healthcare providers, with the healthcare provider /service user relationship having an inbuilt power imbalance potentially affecting communication [3]. However, while research recognises culturally responsive communication as essential within healthcare, it is not seen to be a consistent aspect of healthcare practice.

International literature on culturally responsive communication indicates that healthcare practitioners can find it difficult to achieve culturally responsive communication due to the perceived complexity and indeterminate nature of the concept of culture [10]. Researchers agree that there is no particular definition of culture [3, 5, 10–12]. Betancourt, Green and Carrillo [13] describe culture as a system of beliefs, values, rules and customs shared by a group and used to interpret experiences and direct patterns of behaviour. Anderson et al. [14] define culture as integrated patterns of human behaviour including the language, thoughts, customs, beliefs and values of racial, ethnic, religious or social groups. O’Toole [3] describes culture as the learned patterns of perceiving, interpreting and adapting to the world. Additionally, culture is seen as a dynamic constantly evolving concept [3, 5]. None of these descriptions are contradictory; all suggesting that culture relates to group membership and an unconscious expression of similarities [3].

In order to explore culturally responsive communication in the literature, alternative terms such as ‘transcultural’ and ‘cross-cultural’ were used to examine the concept. Various terms, such as ‘appropriate’ ‘competent’, ‘congruent’, ‘responsive’, ‘safe’ and ‘sensitive’, are used interchangeably with ‘responsive’. ‘Responsive’ was selected as the term used in this study. The commonly used term ‘competence’ implies the need for healthcare practitioners to become completely proficient in an unfamiliar culture [15]. However, it is difficult to be completely aware of all cultural nuances unless ‘growing up’ in the particular culture. The term responsive implies the ability to accommodate the cultural needs of the service user rather than being able to function without error in their culture. Thus, culturally responsive communication can be defined as communicating with awareness and knowledge of cultural differences and attempting to accommodate those differences. This involves respect and an understanding that socio-cultural issues such as race, gender, sexual orientation, disability, social class and status can affect health beliefs and behaviours [3, 6, 7]. Therefore providing person-centred healthcare requires culturally responsive communication [3]. However, international literature suggests inconsistencies in healthcare practitioner knowledge of the core components required to achieve culturally responsive communication.

The literature reviewed and listed above was predominately from international medical and nursing settings due to the limited amount of research relating to cultural communication in the Australian context. This was one of the two limitations of this review. The other was the focus of the reviewed literature on culture relating to racial/ethnic minorities, to the exclusion of disability, gender, age, sexual orientation and religious cultures.

The Australian Government in policies and legislation, including the safety and quality frameworks, and the 2011 Australian Communication Healthcare Charter mandates culturally responsive communication [16–18]. Therefore, this study aims to present a brief overview of the literature (for all healthcare professions), in Australia, exploring the perceived realities, components and effects of this style of communication. The scope of this review considers culture as including ethnicity or race, disability, gender, age, sexual orientation and religion. To the authors knowledge, there are no previous reviews of this kind.

The objective of this rapid review was to evaluate and use the current available evidence to answer the research questions relating to the perceptions of and the requirements for achieving culturally responsive communication and the effects of such communication in Australian healthcare.

The resultant research questions relate to Australian healthcare and are seeking evidence relating to:

1. What are the perceived realities of culturally responsive communication in Australian healthcare?
2. What is required to achieve culturally responsive communication in Australian healthcare?
3. What are the possible effects of culturally responsive communication?

The primary outcomes will be the incidence and effect of culturally responsive communication in Australian healthcare settings. This can be used to inform policy and create training modules to further the use of this type of communication in healthcare.

## Methods

### Study design

A rapid review uses simplified systematic review processes. These processes typically produce a synthesis of information in a shorter period of time [19]. Rapid review methodology produces a timely combination of evidence by limiting scope (i.e. search terms and inclusion criteria) and aspects of synthesis (i.e. data extraction and bias assessment), preferably with minimal impact on quality [19–22]. Steps taken to make this review rapid are shown in Additional file 1. A rapid review was undertaken over a nine-week period from late August to October 2018 using the knowledge to action evidence summary approach to guide the process [20].

### Search strategy

Medline, Cinahl and Proquest electronic databases were searched using Medical Subject Headings (MeSH) terms and keywords relating to culturally responsive communication in healthcare (see Table 1 for an example). The literature search was limited to articles published in the English language. The reference lists of all included articles were manually scanned for additional relevant literature.

**Table 1:** Draft Medline search strategy used to identify relevant articles on culturally responsive communication

1	(cultur* adj3 (competen* or communicat*)).mp. or Culturally Competent Care/ or Culture/ [mp = title, abstract, original title, name of substance word, subject heading word, floating sub-heading word, keyword heading word, protocol supplementary concept word, rare disease supplementary concept word, unique identifier, synonyms]
2	Cultural Competency/
3	1 or 2
4	Occupational Therapy/
5	Occupational Therapists/
6	Nursing Care/ or Nursing Staff, Hospital/ or Nursing/
7	Allied Health Personnel/ or health services/
8	allied health occupations/ or occupational therapy/ or physical therapy specialty/ or speech-language pathology/
9	(nurs* or doctor* or midwife or midwives or allied health or aged care worker* or health worker* or health employee* or health personnel or health professional* or OT or occupational therap* or physiotherap* or dietitian* or nutritionist* or podiatrist* or radiographer* or speech pathologist* or physical therapist* or dental therapist* or social worker* or psychologist* or sonographer* or rehabilitation counsellor* or perfusionist* or osteopath or exercise physiologist* or genetic counsellor* or audiologist* or Orthoptist* or Orthotist* or prosthetist* or Counsellor* or Pathologist* or oral health therapist* or physician*).tw,kw.
10	4 or 5 or 6 or 7 or 8 or 9
11	3 and 10
12	australia*.mp. or exp. AUSTRALIA/
13	11 and 12
14	limit 13 to yr = “2008 -Current”

### Eligibility criteria

The inclusion criteria included peer-reviewed articles discussing culturally responsive communication in Australian healthcare settings published between 2008 and 2018. Only peer-reviewed articles were included in the study to ensure reliable results. All articles were evaluated using the AMSTAR checklist for systematic reviews [23]; the McMasters qualitative critical review form [24]; and the mixed method appraisal tool [25]. Articles were considered appropriate quality and included in this review if they contained transparency about the rigor in the design, implementation and reporting of their research. Articles not published in English and articles deemed to have limited quality were excluded from the study.

### Study selection

**Fig. 1:**
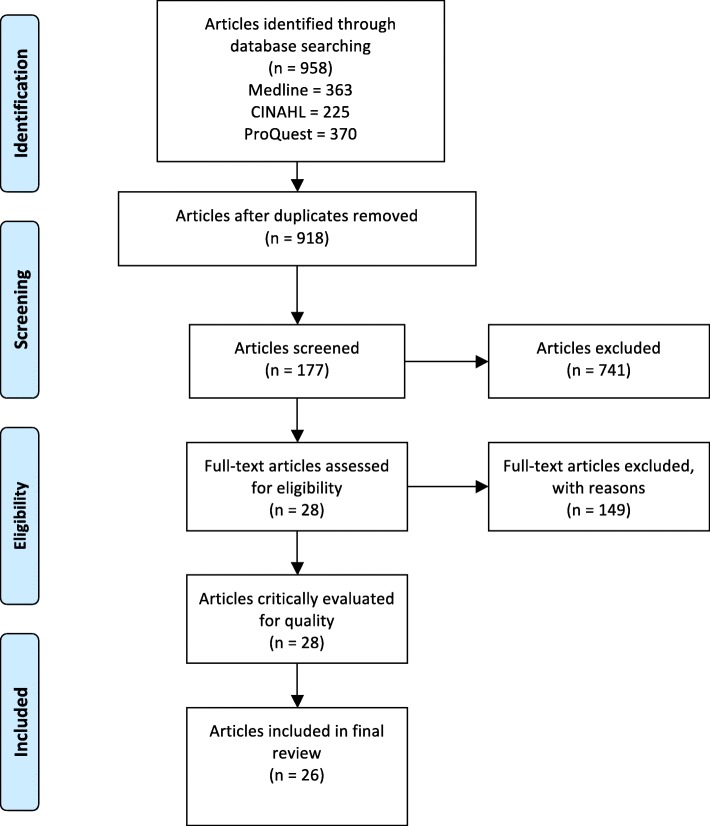
Article selection process

A single reviewer performing title and abstract screening against the inclusion criteria screened results from the electronic database searches. The content of the selected articles was then analysed against the research questions to identify the final articles for review. All articles identified in the database search were screened using the selection process as shown in Fig. 1.

### Quality assessment

Quality assessment appraisals were undertaken by a single reviewer to maintain consistency in appraisal of the identified articles. The AMSTAR checklist is an 11 item measurement tool with good face and construct validity, used to assess the methodological quality of systematic reviews [23]. This checklist evaluates the overall research process, the relevance and details of the research questions and associated methods; inclusion and exclusion criteria, risk of bias (including small study bias), appropriate statistical methodology, consideration of funding and conflict of interest. The McMasters qualitative critical review form contains 21 questions to guide evaluation of qualitative articles [24]. This review form evaluates the study background, purpose, research questions and associated design, along with study selection processes, quality of data management, relevance of conclusions and overall rigour. The mixed method appraisal tool is designed to appraise the methodological quality of mixed method studies retained for systematic reviews [25]. These review procedures were selected to facilitate the rapid appraisal of relevant literature. The strength of the body of evidence cumulated in this review will be assessed using the AMSTAR checklist [23]. The results of the AMSTAR checklist can be seen in the following discussion.

### Synthesis of review

Qualitative findings from the included publications were synthesized using tables and a narrative summary by a single reviewer. The review of identified articles used the definition of culture mentioned above, and considered the occurrence of repeated ideas and relevance to the research questions in each article. The recurring ideas were grouped into themes and sub themes. Data extracted included demographic information, methodology, aims and relevant findings (see Table 2: Details of reviewed articles).

**Table 2:** Summary of the included articles, ordered chronologically, from most to least recent, and alphabetically within years

Ref	Author/Year	Type of study	Setting	Sample	Aims	Relevant findings
[26]	Hughson, Marshall, Daly, Woodward-Kron, Hajek & Story (2018)	Qualitative	Medicine - Maternity	7 midwives, 5 obstetricians, 5 physiotherapists, 1 social worker and 1 occupational therapist working with CALD service users	Identify health literacy issues when providing maternity care to CALD women, and the strategies needed for health professionals to collaboratively address these issues	Health professionals reported a lack of certainty as to whether the information they were trying to communicate was adequately comprehended, low health literacy of the service users and competing cultural models of health barriers to effective culturally responsive communication
[27]	Jennings, Bond & Hill (2018)	Systematic review	Non-specific healthcare	65 reports on Indigenous healthcare access	Explore Indigenous narrative accounts of healthcare access within qualitative research papers, to better understand Indigenous views on culturally safe healthcare and health communication represented in that literature	Indigenous service users valued informal ‘talk’ and the use of simplified language within healthcare interactions as it fostered feelings of trust, strengthened engagement and produced positive outcomes
[28]	Mollah, Antoniades, Lafeer & Brijnath (2018)	Qualitative	Mental health	4 counsellors, 6 psychologists, 5 nurses and 2 social workers working with CALD communities	Document frontline mental health practitioners understanding of cultural competence and to identify, from their perspective, what helped or hindered them to deliver culturally competent mental healthcare in their daily practice	Healthcare providers reported not achieving effective cross-cultural communication was due to a lack of access to, reliability of and use of interpretive services. Among the participants who felt they were achieving culturally responsive communication, many of the communication styles described tended to homogenize ethnic differences between practitioner and patient but highlighted ethnic differences from the mainstream community
[29]	Xiao, Willis, Harrington, Gillham, De Bellis, Morey & Jeffers (2018)	Qualitative	Nursing – Aged care	56 aged care workers and 30 Culturally And Linguistically Diverse (CALD) aged care residents and their family members	Critically examine how staff and residents initiated effective cross-cultural communication and social cohesion that enabled positive changes to occur	Cultural humility, a collaborative approach and organizational support is critical to achieving effective cross-cultural communication
[30]	Smith, Fatima & Knight (2017)	Mixed methods	Non-specific healthcare	24 healthcare providers and 54 Aboriginal service users	Explore the views of key stakeholders on cultural appropriateness of primary healthcare services for Aboriginal people	A practice level gap exists between healthcare workers and Aboriginal service users’ perceptions for the provision of culturally sensitive services delivery
[12]	Truong, Gibbs, Paradies & Priest (2017)	Qualitative	Allied health	14 community healthcare providers working with CALD communities	Explore the multi-level aspects of cultural competence from the perspectives of healthcare service providers in the community health context	Reflexivity at both individual and organizational levels is necessary in order to deliver services that are responsive to local community needs
[31]	Truong, Gibbs, Paradies, Priest & Tadic (2017)	Qualitative	Allied health	27 CALD community health service users	Explore the positioning of cultural competence within community health from multiple perspectives	Healthcare professionals reported barriers to achieving effective cross-cultural communication as the services users’ level of English-speaking proficiency and their understanding the language of the ‘health system’. Service user participants did not feel as though they experienced any language difficulties and were offered interpreters as needed, however found it easier to communicate with staff that shared a similar cultural background
[32]	Watts, Meiser, Zilliacus, Kaur, Taouk, Butow, Kissane, Hale, Perry, Aranda & Goldstein (2017)	Qualitative	Medicine - Oncology	12 medical oncologists, 5 radiation oncologists and 21 oncology nurses working with CALD cancer patients	Identify the systemic barriers encountered by oncology health professionals working with patients from ethnic minorities to guide the development of a communication skills training program	Health professionals expressed a need for training in cultural awareness and communication skills with a preference for face-to-face delivery. A lack of funding, a culture of “learning on the job”, time constraints and the belief that any single culture was too diverse for cultural training to be beneficial were systemic barriers to training
[33]	Henderson, Barker & Mak (2016)	Qualitative	Nursing	19 clinical facilitators, 5 clinical nurses and 10 nursing students working with CALD communities	Explore the experiences of clinical nurses, nurse academics and student nurses regarding intercultural communication challenges	Strategies participants used to mitigate challenges included resorting to cultural validation through alliance building, proactively seeking clarification and acquiring cultural awareness knowledge
[34]	Olaussen & Renzaho (2016)	Systematic review	Allied health	11 papers reviewed – reporting on migrants with disabilities	Examine the challenges of providing service to migrants with disability, healthcare providers level of cultural competence and document components of the cultural competence framework required to reduce disability-related health inequalities	Healthcare professionals perceived themselves as being culturally competent, whereas migrants with disabilities and their families felt as though their needs were not being adequately addressed due to cultural misunderstandings and disrespect of cultural values
[35]	Valibhoy, Kaplan & Szwarc (2016)	Qualitative	Mental health	16 young people from refugee backgrounds	Explore the perceptions of young people from refugee backgrounds how they accessed mental health services, disclosing personal problems, barriers and facilitators to engagement with clinicians and recommendations to improve services	Participants valued accessible practitioners who combined content expertise with interpersonal qualities to make the person feel listened to, responded to and recognized
[36]	Watt, Abbott & Reath (2016)	Qualitative	Medicine – General Practitioner (GP) Registrars	43 GPs & Registrars working with CALD Aboriginal and CALD clients	Explore the ways in which GP registrars are currently developing cultural competence	Registrars report there is no common approach to cross-cultural training. Exposure to diversity appears to be an important way in which cultural competency is developed
[37]	O’Connor, Chur-Hansen &Turnbull (2015)	Qualitative	Mental health	8 psychologists working with Aboriginal clients	Identify the professional skills and personal competencies that enable effective service delivery for Indigenous clients, particularly those aged 12–25	Professional skills needed to achieve culturally responsive service delivery with Aboriginal service users are collaboration, flexibility, commitment to ongoing learning and community engagement and consultation. Personal characteristics include self-reflection, welcoming nature, openness and cultural understanding
[38]	Von Doussa, Power, McNair, Brown, Schofield, Perlesz, Pitts & Bickerdike (2015)	Qualitative	Allied health	32 healthcare workers and 13 same-sex attracted parents	Explore barriers and facilitators to healthcare access for same-sex attracted parents and their children	Healthcare workers and same-sex attracted parents agreed that the workers lacked confidence and knowledge using appropriate and inclusive language to acknowledge the persons family situation
[39]	Wilson, Magarey, Jones, O’Donnell & Kelly (2015)	Qualitative	Allied health	35 non-Aboriginal health professionals working with Aboriginal services users	Explore the attitudes and characteristics of non-Aboriginal health professionals working in Aboriginal health	The attitudes and characteristics of non-Aboriginal health professionals working in Aboriginal health vary and can be considered across a range of groups. Self-reflection is critical for health professionals to address their own assumptions and bias that
[40]	Abbott, Reath, Gordon, Dave, Harnden, Hu, Kozianski & Carriage (2014)	Qualitative	Medicine – GP	71 GP supervisors and 4 medical educators working with Aboriginal service users	Examine the confidence and skills of non-Indigenous GP supervisors in providing feedback to a GP Registrar consulting with an Aboriginal patient	GP supervisors lacked confidence in providing guidance on cross-cultural communication with Aboriginal service users. GP registrars and supervisors felt they lacked specific training and relied on generic communication and consultation skills
[41]	Farley, Askew & Kay (2014)	Qualitative	Non-specific healthcare	20 GPs, 5 practice nurses and 11 administrative staff working with newly arrived refugees	Explore the experiences of primary healthcare providers working with newly arrived refugees in Brisbane	Healthcare providers identified lack of funding, appropriate resources and language barriers as the reason for not achieving effective culturally responsive communication. Healthcare providers reported trying to overcome these barriers by learning basic greetings, making longer appointment times and accessing external supports, such as language classes
[42]	Kendall & Barnett (2014)	Qualitative	Allied health	34 Indigenous health workers and community elders and 5 non-Indigenous health workers	Explore the factors contributing to the underutilization of health services by Aboriginal people	Services users often described the healthcare providers communication styles as an abrupt series of questions or demands followed by the rapid transfer of incomprehensible medical knowledge. Effective and respectful communication allowed Indigenous service users to feel informed and empowered to make knowledgeable decisions
[43]	Nielsen, Foster, Henman & Strong (2013)	Qualitative	Medicine	20 service users diagnosed with chronic pain	Examine the healthcare experiences of people with chronic pain and focuses discussion on the impact that institutional and cultural factors can have on individual experience	Problematic patient-provider communication, such as speaking too ‘clinically’ and ‘talking at’ rather than to can negatively affect the care received and health outcomes of a person living with chronic pain
[44]	Woolley, Sivamalai, Ross, Duffy & Miller (2013)	Qualitative	Medicine – Graduate	13 Indigenous health professionals, Elders and community members	Explore Indigenous peoples’ perspectives regarding desired attributes they would want to see in graduate doctors who choose to practice in their remote community	Effective communication with Indigenous service users and in remote communities required graduate doctors to have appropriate clinical skills, medical knowledge, knowledge about how local health systems operate and familiarity with significant Indigenous health issues
[45]	Gill & Babacan (2012)	Qualitative	Non-specific healthcare	Number and type of participants not specified	Report findings of a major review of one of Australian state health systems cultural and linguistic diversity, cultural competence requirements, minimum standards and benchmarks	The concept of cultural competence was not well defined. A whole-organization approach at all levels of the system is needed to achieve culturally competent communication and care
[46]	Komaric, Bedford & van Driel (2012)	Qualitative	Allied health	50 CALD service users and 14 healthcare providers	Describe the challenges people from CALD communities and their treating healthcare providers face regarding treating and preventing chronic disease and what barriers they experience and perceive with regard to access to health services	The provision of adequate interpretive services was identified by healthcare providers and services users as a means to increase satisfaction with care, however recognized it as an overly simplistic solution
[47]	Mitchison, Butow, Aldridge, Hui, Vardy, Eisenbruch, Iedema & Goldstein (2012)	Qualitative	Medicine - Oncology	73 CALD cancer patients and their relatives	Explore communication of prognosis with migrant cancer patients and their families	Services users from all ethnicities preferred their prognostic information to be delivered in a caring and personalised manner from an authoritative oncologist
[48]	Kaur (2009)	Qualitative	Social work	66 child protection caseworkers working with CALD communities	Examine caseworker perceptions of ‘culturally sensitive’ practice when working with CALD communities	Recognition and acknowledgement of the persons cultural identity, cultural values, languages, community and religion are critical to achieving effective communication
[49]	Johnstone & Kanitsaki (2008)	Qualitative	Non-specific healthcare	145 healthcare workers self-identified as being from different ethno cultural backgrounds	Explores the idea that racial and ethnic disparities in healthcare may be expressive of un-acknowledged practices of cultural racism	The language difference and English language proficiency of the service user was used as a social marker to classify and categorise patients and had a significant influence on how they were treated by attending staff. This language prejudice was found as a profound failure in and a barrier to communication
[50]	*Renzaho (2008)*	*Qualitative*	Allied health	50 healthcare workers and 100 CALD service users	Document how service providers identify and develop services to meet the needs of CALD communities and assess CALD clients’ experiences with the service providers	Service providers have limited approaches to the provision of CALD services, tending to adopt a “one-size-fits-all” models of delivery

*CALD* Culturally And Linguistically Diverse, *GP* General Practitioner

## Results

A total of 958 articles retrieved from electronic databases were screened for inclusion (see Fig. 1 for article selection process). Overall, 26 articles were included in the review (article characteristics are listed in Table 2). There are 23 qualitative studies, 2 systematic reviews and 1 mixed method study considered appropriate for this rapid review. The settings for the studies included: allied health (*n* = 8), medicine (*n* = 7), non-specific healthcare (*n* = 5), mental health (*n* = 3), nursing (*n* = 2) and social work (*n* = 1). The setting was considered non-specific if the study was in the context of a hospital or a combination of multiple medicine and allied health professions. The populations studied for the reviewed articles, using the abovementioned understanding of culture, were: culturally and linguistically diverse (CALD) and/or refugee (*n* = 15), Aboriginal and Torres Strait Islander (*n* = 7), non-specific diverse populations (*n* = 2), people with chronic pain (*n* = 1) and lesbian, gay, bisexual, transgender, intersex (LGBTI) (*n* = 1). If the cultural origin of the service user was not identified the population was classified as non-specific. Only 4 of the 26 included articles specifically explored culturally responsive communication. The other 22 articles discussed this style of communication within the context of culturally responsive care and/or practice. Approximately 73% of the healthcare provider or consumer participants were female in the 19 articles specifically reporting participant characteristics.

This review aimed to explore three major themes relating to culturally responsive communication: perceived realities, aspects of and its effects. For each of these themes, there were identified sub-themes, reported below.

### Perceived realities of culturally responsive communication

#### Healthcare practitioner perceptions and beliefs

The results of this review indicate that healthcare practitioners lacked confidence in their ability, skills or knowledge to achieve effective culturally responsive communication [36, 38, 40, 45, 48]. This resulted in many healthcare practitioners adopting a generic ‘one-size-fits-all’ style of communication, thereby displaying attitudes of ‘cultural blindness’ [12, 28, 34, 40, 50].

#### Service user perceptions

The perceptions of the service users indicated that healthcare practitioners style of communication was not culturally responsive [27, 30, 34, 35, 38, 42, 49, 50]. Service users felt that healthcare practitioners presented as sceptical, authoritarian and patronising [27, 42, 43, 49] using complicated explanation with excessive jargon [27, 42, 44].

#### Training and education

The results indicated that many healthcare practitioners felt they did not receive sufficient, if any, formal training on how to achieve culturally responsive communication [12, 28, 30, 33, 36–38, 48]. Many healthcare practitioners presented as positive and motivated to further their education in culturally responsive communication [12, 26, 28, 29, 33, 38, 41, 46], however did not feel supported to do so by their employer, or know where to access such training [12, 33, 38, 41]. All the reviewed literature recommended the need for further formal training in the concept of culturally responsive care and communication, as well as requiring a reliable evaluation method to be used within services.

#### Workplace factors

There are various workplace factors facilitating the achievement of culturally responsive communication. The literature suggests that these factors were often absent from many healthcare workplaces. Availability of resources and literature in relevant languages with appropriate graphics is also a factor indicating a commitment to culturally responsive communication [32, 34, 37, 38, 40, 41, 43, 45, 46, 49, 50]. The employment of culturally diverse staff reflecting the represented cultures of its service users [26, 28–30, 37, 40, 45, 46], along with the availability and use of quality interpreter services contribute to the ability to achieve culturally responsive communication [12, 26, 28, 29, 32, 34, 41, 45, 46, 49, 50]. The literature revealed that healthcare practitioners often cited interpreters as the cause of miscommunications, affecting their inability to achieve culturally responsive care and communication [12, 26, 28, 32, 34, 46].

### Requirements of culturally responsive communication

The essential components of culturally responsive communication identified in the reviewed literature were categorised into three sub-themes. See Table 3 for the differences in opinion between the healthcare practitioners and service users in relation to these sub-themes:

**Table 3:** Summary of themes considering healthcare practitioner and service user perspectives

	Sub-theme	Perceptions of healthcare practitioners	Perceptions of service users
*Required characteristics of the healthcare practitioner communicator*	Reflexivity	[12, 28, 36–41, 44, 45, 50]	[27]
	Flexibility	[28, 37, 41, 45]	[44]
	Self−/other-awareness	[28, 36, 37, 40]	
	Respectful	[12, 38, 40, 48]	[12, 34, 35, 42, 44]
	Trustworthy		[27, 34, 44]
	Honest and transparent	[37, 38]	[34, 38, 42, 44]
	Non-judgemental	[37, 38]	[27, 35, 38]
	Willing to learn	[12, 32, 33, 40, 41, 46]	[44, 46]
*Required foundational communication skills*	Ability to listen	[32, 41]	[27, 34, 35, 42–44]
	Checking understanding	[32, 33, 40]	[27, 44]
	Inclusion and/or acknowledgement of family	[41]	[34, 38, 50]
	Use of simplified, inclusive language	[12, 38]	[27, 30, 34, 38, 42, 44]
*Required contextual factors*	Diversity in staff	[26, 28, 29, 37, 40, 46]	[29, 30, 46]
	Access to culturally appropriate resources and literature	[29, 32, 38, 40, 41, 49]	[34, 38, 50]
	Availability, quality and use of interpreter services	[12, 26, 28, 41, 46, 50]	[12, 34, 46]

#### Required characteristics of the healthcare practitioner communicator

The characteristics that a healthcare practitioner must display to achieve culturally responsive communication include: self-reflection and reflexivity [12, 27, 28, 36–41, 44, 45, 50], flexibility [28, 37, 41, 44, 45], self- and other- awareness [28, 36, 37, 40], being respectful [12, 34, 35, 38, 40, 42, 44, 48], being trustworthy [12, 27, 34, 44], being honest and transparent [34, 37, 38, 42, 44], being non-judgmental [27, 35, 37, 38] and have a willingness to learn [12, 32, 40, 41, 44, 46].

#### Required foundational communication skills

Specific communication skills and behaviours required to achieve effective cross-cultural communication include: ability to listen [27, 32, 34, 35, 41–44], clarifying understanding [27, 32, 40, 44], inclusion and/or acknowledgement of family [34, 38, 41, 50], limiting the use of jargon [12, 27, 30, 34, 38, 42, 44] and using inclusive language [12, 27, 30, 34, 38, 42, 44].

#### Required contextual factors

Contextual factors beyond the control of individual healthcare practitioners facilitating culturally responsive communication include: diversity in staff [26, 28–30, 37, 40, 45, 46], access to culturally appropriate resources and literature [29, 32, 34, 38, 40, 41, 45, 49, 50], and availability, quality and use of interpreter services [12, 26, 28, 34, 41, 45, 46, 50].

### Effects of culturally responsive communication

The effects of achieving culturally responsive communication include: improved health outcomes and decreased health disparities of marginalised populations [27, 34, 41, 43–46, 49, 50], increased access to and utilisation of mainstream healthcare services [12, 27, 30, 34, 37, 38, 40, 41, 44, 50], increased mutual understanding resulting in increased quality of care [30, 41–46], positive therapeutic relationships and rapport between service users and healthcare practitioners [27–29, 33, 34, 37, 40, 41, 48, 49], increased service user trust and satisfaction with the clinical encounter [27, 30, 35, 37–39, 43, 45–47], reduced stereotyping [12, 33, 40], and increased healthcare practitioner knowledge and confidence [26, 29, 31, 33, 40, 41].

## Discussion

This review found that there is limited evidence available reporting specifically on culturally responsive communication in Australian healthcare settings. The results of this review found evidence about the reality, components and effects of this style of communication. However, it was predominately discussed in international literature outside Australia, within the context of culturally responsive practice and/or care, demonstrating limited understanding of the need for culturally responsive communication to achieve this type of care. Additionally, there was a focus in the literature on the barriers to achieving this style of practice and/or care, rather than discussing or measuring its success. Findings from this review highlight the difficulties of researching the existence of culturally responsive communication in all settings due to the difficulties of recognising it in healthcare. This could be due to the ambiguous nature of the concept and the difficulty of defining ‘culture’, thereby creating various interpretations of the concept [5, 10]. In addition, there is no formal assessment to measure the success of individual healthcare practitioners in using culturally responsive communication. Instead, the literature relied on healthcare practitioner self-reports about the quality of their culturally responsive communication. It is interesting to note that these healthcare practitioner self-reports were often contradictory to the perceptions of the service users.

The literature revealed that healthcare practitioners felt that they achieved effective culturally responsive communication despite lacking confidence in the knowledge and skills relating to this style of communication. In contrast, the service users reported that healthcare practitioner styles of communication were patronising, lacked a nuanced approach to cultural sensitivity and used excessive jargon. This discrepancy may relate to limited healthcare practitioner reflection about their communication skills and/or a tendency towards ethnocentrism hindering their respect and appreciation of the perspective of service users [51]. This tendency for the healthcare practitioners to view themselves and their communication styles positively may be in part due to the ethnocentric attitudes often typical of a western healthcare model [52]. It is the responsibility of the healthcare practitioners to regularly engage in honest self-reflection to challenge their assumptions and critically examine their role within cross-cultural interactions and the effect of their communication style upon the health outcomes of service users [3, 36, 38, 39, 45, 50]. This requires deconstruction of ethnocentric values affecting communication and care within the healthcare system [3, 51].

The findings of this review highlighted a focus on person-centred care for all healthcare practitioners. This focus revealed a belief of the importance of person-centred care over culturally responsive communication. The belief of the importance of being person-centred over and above being culturally responsive suggests limited understanding of the relationship between person-centred care and culturally responsive communication. In reality to be culturally responsive is to be person-centred in healthcare [3]. A few articles [28, 45] presented the belief that culturally responsive communication was embedded within policies to achieve minimum standards rather than being a requirement of effective healthcare.

A healthcare practitioner must communicate with respect, always respectfully acknowledging and accommodating the cultural aspects of the person if they aim to achieve person-centred practice [3]. However, no one person can know everything about every culture [3]. Therefore, acknowledging and accommodating the expertise of the service user, their family and/or community about their life, culture and needs, instead of the healthcare practitioner assuming the ‘expert-educator’ role is essential [51]. Communicating without accommodating the unique culture of each person results in healthcare practitioners adopting a generic style of communication resulting in ‘treating everyone the same’ often called ‘cultural blindness’. Cultural blindness can potentially lead the healthcare practitioner to unconsciously ‘favour’ the most assimilated service user therefore overlooking opportunities to reduce health disparities of culturally diverse individuals, but especially marginalised groups [40].

The results from this review revealed that despite expectations of some employers to attend cultural ‘competence’ training, healthcare practitioners did not feel as though they have received enough training to achieve effective culturally responsive communication. Healthcare practitioners consistently reported desire and motivation to continue their learning about how to communicate in a culturally responsive manner. However, external barriers, such as systemic racism, funding issues, and increasing administration duties and accountability, and thereby decreasing the time of face-to-face interactions, were often cited as the reason for not accessing further training in culturally responsive communication. In addition, when considering workplace factors affecting culturally responsive communication, limited time and funding for resources were often listed as barriers. This suggests the need for change of policy in both organisations and at government levels.

The literature revealed that both healthcare practitioners and service users adequately understand the required personal factors and communication behaviours to achieve effective culturally responsive communication in healthcare. These factors mentioned above, include self-reflection and reflexivity, flexibility, self- and other- awareness, being respectful, worthy of trust, being honest and transparent, non-judgmental and willing to learn. However, a discrepancy between the opinions of healthcare practitioners and service users was revealed in the personal factor of self-reflection and reflexivity. This personal factor was almost exclusively cited by healthcare practitioners, with only one service user mentioning it as a requirement. This could be due to tertiary training emphasising the need for self-reflection and reflexivity in healthcare communication. Another discrepancy was the service users reporting a need for healthcare practitioners to limit their use of jargon, with healthcare practitioners not appearing to be aware of the effects of professional jargon. The use of jargon in healthcare communication can cause confusion and disempowerment if the service user has no knowledge, understanding or experience of the terminology [3, 27]. An additional difference was the need to be more inclusive of family during healthcare. Self-reflection may assist health practitioners to identify their beliefs regarding family involvement. If the health practitioner grew up in an individualistic, western culture, they may not recognise the importance of involving service user families and/or communities in all healthcare communication. Only two of the reviewed articles [29, 40] identified the personal factor of humility as a requirement to achieve culturally responsive communication. Humility allows the healthcare practitioner to accommodate cultural differences and to take responsibility for inappropriate communication [3].

Another factor affecting achievement of culturally responsive communication was healthcare practitioner perceptions that interpreters cause miscommunications during cross-cultural healthcare encounters. This may reflect limited training of healthcare practitioners in how to effectively use interpreter services. There is limited formal training in use of interpreter services in many healthcare services with this training not always being readily available to all healthcare professions [12, 26, 34, 41, 46, 50]. In addition, the training and availability of appropriate interpreter services varies depending on location.

The literature indicates the positive effects of culturally responsive communication upon both the healthcare process and related outcomes. These positive outcomes relate to the fundamental right of every human to experience health [51, 53] as well as satisfaction with the healthcare process [45]. The satisfaction of service users from culturally responsive communication while experiencing healthcare result in adherence to treatment protocols, retention and understanding of relevant information and improved health [28, 30, 31, 34, 43, 45, 50]. This also produces increased satisfaction for the healthcare practitioner and their employers. Despite these overall positive outcomes of culturally responsive communication, the limited Australian literature relating to this style of communication suggests:

- a lack of awareness of the importance and positive outcomes of culturally responsive communication or
- a focus on the barriers rather than the relevance or
- limited commitment or motivation at policy and organisational levels and thus willingness to fund and support culturally responsive communication in practice.

Overall, this indicates the need to expand the concept of culturally responsive communication from the rhetoric of policy, legislation and literature and into the reality of everyday healthcare practice.

### Strengths and limitations of the study

A key strength of this rapid review is its identification of the limited research into this area of healthcare communication. Of the 26 articles included in the final review, only 4 specifically explored culturally responsive communication rather than practice and/or care. The identification of this research gap is significant, especially considering the well-known effects of achieving this style of communication. There are limitations affecting the findings of this rapid review (see Additional file 1 for shortcuts taken to make this review rapid). Limiting the search to three databases may introduce publication bias thereby possibly omitting potentially relevant publications [21]. A single reviewer, to ensure consistency and appropriate use of limited time, may result in reviewer bias also a possible limitation of this rapid review. The quality of the research included in the review varied, which may introduce limitations in the validity and reliability of the findings. The majority of articles included in the review were qualitative studies with a small sample size, potentially limiting the generalisability of the results. The results of this review may be considered to contain a gender bias, with approximately 73% of participants being female in the 19 articles specifically reporting participant characteristics. Additionally, the included literature focussed mainly on the cultural aspects of CALD and Indigenous populations, with a limited focus on disability, gender, age, sexual orientation and religious cultural aspects.

## Conclusion

Overall, the results relating to the realities of culturally responsive communication in Australian healthcare are disappointing. Findings suggest a need for healthcare practitioners to commit to ongoing reflective practice to honestly evaluate the cultural responsiveness of their communication style. There is also a need for further training on how to recognise and achieve culturally responsive communication, as well as the development of a formal assessment tool to measure the success of individual healthcare practitioners with this style of communication. In addition, all levels of health organisations need to recognise and take responsibility for fostering a culture of reflection about and achievement of culturally responsive communication within their service. In combination, such efforts will improve healthcare services for all service users whether from non-marginalised or marginalised groups in Australian society.

## Supplementary information

**Additional file 1.** Steps taken in this review to make it a rapid and quality assessment.

## Data Availability

All datasets supporting the conclusions of this article are included within this article and its additional files. The Occupational Therapy department of the University of Newcastle, Australia is the Sponsor, meaning that it is responsible for the data.

## Abbreviations

AMSTAR: A MeaSurement Tool to Assess Reviews
CALD: Culturally And Linguistically Diverse
GP: General Practitioner
LGBTI: Lesbian, Gay, Bisexual, Transgender, Intersex
MeSH: Medical Subject Headings
