# Supplementary material for: Exploring the incidence of culturally responsive communication in Australian healthcare: the first rapid review on this concept
Source: BMC Health Serv Res. 2020 Jan 7;20:20. doi: 10.1186/s12913-019-4859-6 (PMC6947994; doi:10.1186/s12913-019-4859-6)
Supplement: Supplementary file 1 — Additional file 1. Steps taken in this review to make it a rapid and quality assessment. [file 12913_2019_4859_MOESM1_ESM.docx]

**Additional file 1** Steps taken in this review to make it a rapid and quality assessment

- One reviewer screened titles and abstracts
- One reviewer extracted data with checking by a second reviewer
- Data extraction limited to recurring key characteristics and results
- Exclusion of grey literature
- Limit placed on language of publications – English only
- Limit placed on location of research – Australia only
- Narrow time frame of search – studies published between 2008-2018
- Narrative synthesis only
- Publication bias not assessed, although no clear methods available for qualitatively assessing publication bias
